# Procedural Sedation in the Emergency Department – An Observational Study: Does Nil Per Os Status Matter?

**DOI:** 10.5811/westjem.18561

**Published:** 2025-01-15

**Authors:** Brendan Peterson, Amy S. Nowacki, Alexander Ulintz, Sharon E. Mace

**Affiliations:** * Ohio State University, School of Pharmacy, Columbus, Ohio; † Cleveland Clinic Lerner College of Medicine of Case Western Reserve University, Cleveland, Ohio; ‡ Lerner Research Institute, Department of Quantitative Health Sciences, Cleveland Clinic, Cleveland, Ohio; § Ohio State University, Department of Emergency Medicine, Columbus, Ohio; ∥ Cleveland Clinic, Department of Emergency Medicine, Cleveland, Ohio

## Abstract

**Introduction:**

Procedural sedation (PS) is commonly performed in the emergency department (ED). Nil per os (nothing by mouth) (NPO) guidelines extrapolated from standards for patients undergoing elective procedures in the operating room have been applied to ED PS patients. There has been no large study of ED PS patients comparing differences in adverse events and PS success rates based on NPO status.

**Methods:**

From a cohort of consecutive ED PS patients of all ages in the 20 EDs of one hospital system—one quaternary ED, four tertiary EDs, six community hospital EDs, one rural ED, two pediatric EDs, and six freestanding EDs in two states in the Midwest and South—we conducted a retrospective analysis on a prospective database over 183 months from April 2000–June 2015. Primary outcome was the incidence of side effects and complications, which comprised the adverse effects. The side effects were nausea, vomiting, itching/rash, emergence reaction, myoclonus, paradoxical reaction, cough, and hiccups. Complications were oxygen desaturation <90%, respiratory depression (respiratory rate <8), apnea, tachypnea, hypotension, hypertension, bradycardia, and tachycardia. Normal vital signs were age dependent. Secondary outcome was successful sedation defined as completion of the procedure. We examined the association between adverse events and successful sedation with NPO status.

**Results:**

Of 3,274 visits, exact NPO status was known in 2,643 visits. Comparison of NPO <8 hours in 1,388 patients vs ≥ 8 hours in 1,255 patients revealed side effects 5.5% vs 4.5% (*P* = 0.28); complications 11.9% vs 17.7% (*P* < 0.001); adverse events 16.3% vs 21.5% (*P* < 0.001), interventions 4.1% vs 4.4% (*P* = 0.73), and procedural completions 94.3% vs 89.7% (*P* < 0.001). After adjustment for age, sex, transfer status, American Society of Anesthesiology physical status classification, race, primary sedative, multiple sedatives, sedative plus analgesic, and primary analgesic, we found no association between NPO status and side effects (*P* = 0.68), complications (*P* = 0.48), or adverse effects (*P* = 0.26); however, procedural completion rate remained significantly higher for NPO < 8 hours (*P* = 0.007).

**Conclusion:**

A nil per os status ≥8 hours may have similar or worse outcomes than NPO <8 hours, which is contrary to many suggested guidelines. Strict adherence to NPO guidelines in ED procedural sedation patients may not be necessary.

Population Health Research Capsule What do we already know about this issue?

*Procedural sedation (PS) is a common ED procedure. Applying anesthesiology nil per os (NPO) guidelines for elective procedures to ED PS patients has been questioned.*

What was the research question?

*What is the incidence of adverse event and procedural completion rates for patients meeting vs not meeting NPO guidelines?*

What was the major finding of the study?

*NPO <8 vs ≥8 hours: adverse events 16.3% vs 21.5% P < 0.001, procedural completions 94.3% vs 89.7% P < 0.001.*

How does this improve population health?

*NPO ≥8 hours has similar or worse outcomes than NPO <8 hours, contrary to many suggested guidelines. Strict adherence to NPO guidelines in ED PS patients may not be necessary.*

## INTRODUCTION

Emergency department (ED) patients frequently undergo procedural sedation (PS) and analgesia, which is designed to alleviate their pain and anxiety during diagnostic and/or therapeutic medical procedures.^1^ Guidelines regarding fasting prior to performing PS, promulgated by various organizations for general anesthesia, are often followed by clinicians performing PS in the ED.^1^ ^–^ ^5^ This concept has recently been challenged.^5^ There has been some evidence in the pediatric population that adherence to such guidelines does not result in fewer adverse events during ED PS,^6^ ^–^ ^13^ although some of the reports of pediatric PS have involved PS performed in locations other than the ED^6^ ^,^ ^7^ or involved only one sedative instead of a range of sedative agents.^8^ ^,^ ^12^ Such data is lacking for adult ED patients. Our goal in this study was to evaluate the effect of fasting on PS in the ED in all ages of ED patients, including the elderly.

### Importance

Studies in the literature on the effect of fasting on ED PS have focused on the pediatric population, with a surprising lack of studies in adults, including the elderly. A large-scale study of the incidence of adverse events and the need for interventions has not been described, thus representing a large gap in knowledge for a common practice.

### Goals of This Investigation

Our goal in this study was to determine the impact of fasting guidelines on the side effects, complications, and need for interventions during ED PS in patients of all ages.

## METHODS

### Study Design and Setting

This was a cohort study of consecutive patients of all ages undergoing PS in the 20 EDs of one hospital system consisting of one urban, academic, quaternary ED, four tertiary EDs, six community hospital EDs, one rural ED, two pediatric EDs, and six freestanding EDs in two states located in the Midwest and the South. We performed a retrospective analysis on a prospectively collected database over 183 months from April 2000–June 2015. All patients who underwent parenteral PS in the ED, performed by attending emergency physicians (EP) were included. We excluded sedations done outside the ED and/or not administered by EPs.

### Data Collection

A mandatory, four-page, standardized sedation form must be completed by the registered nurse, respiratory therapist, and attending physician on all patients undergoing PS throughout the hospital including the ED. This form includes pre-sedation assessment, post-sedation assessment (including readiness for discharge), and documentation of the PS itself. Documentation of the PS includes the continuous monitoring of vital signs: heart rate, respiratory rate, blood pressure; pulse oximetry, cardiac rhythm, respiratory therapy assessment, including capnography; and patient responses, medication administration, and patient interventions. All sedations including the sedation forms and electronic health record (EHR) notes are reviewed as part of the hospital quality improvement (QI) monthly meeting by a physician-led committee. The members of this committee were not involved in this study but are part of the hospital’s QI process.

We performed this retrospective review with adherence to the 12 methodologic criteria as defined by Worster et al.^14^ Data resulted from an electronic pull of information from the EHR. We did not use abstractors. Therefore, criteria 1, 4, 5, 6, 7, and 8 according to Worster et al were not applicable. The remaining criteria (2, 3, 9, 10, 11, and 12) were met. For criterion 2, case selection criteria were defined a priori. For criterion 3, variables were defined in the methods. For criterion 9, the health record database was described. For criterion 10, all patient visits in the EHR meeting criteria were included. For criterion 11, data used was part of a mandatory standardized sedation form, and the missing data was minimal. As discussed in our study flow diagram, we conducted a complete case analysis. For criterion 12, the institutional review board approved the study.

### Outcome Measures

The primary outcome was the incidence of side effects and complications, which comprised the adverse effects. Side effects were nausea, vomiting, itching/rash, emergence reaction, myoclonus, paradoxical reaction, cough, and hiccups. Complications were oxygen desaturation <90%, respiratory depression with a respiratory rate <8, apnea, tachypnea, hypotension, hypertension, bradycardia, and tachycardia. The normal range of vital signs was age dependent. Successful sedation was completion of the procedure.

### Statistical Analysis

Descriptive statistics of patient demographics and procedures are presented as count (percentage), median (Q_1_–Q_3_), or range. We explored bivariable associations of patient demographics and procedures with NPO status with either a Wilcoxon rank-sum test or a chi-square test, as appropriate. The NPO was originally recorded as a numeric value in patient charts and, thus, we explored NPO status three ways: (1) classified as exact NPO status known or unknown; (2) dichotomized at eight hours to align with clinical care guidelines; and (3) original scale to maintain full detail. We explored associations of NPO status with binary outcome measures using generalized estimating equations models, assuming a compound symmetry correlation structure to accommodate multiple ED visits per patient. This was performed both unadjusted and adjusting for year of visit, patient age group, sex, transfer status, American Society of Anesthesiology (ASA) physical status classification, race, primary sedative, use of multiple sedatives, use of sedative plus analgesic, and primary analgesic. The ASA is used to predict operative risk where ASA 1 is a normal healthy patient; ASA 2 is a patient with mild systemic disease; and ASA 3 is a patient with severe systemic disease that is not life-threatening. The ASA 4 is a patient with severe systemic disease that is a constant threat to life; ASA 5 is a moribund patient who is not expected to survive without the operation.^15^ Reported are the resulting odds ratios, 95% confidence intervals, and associated *P*-values. Similar analyses were conducted to explore the association of NPO status with the need for medical intervention. We used a significance level of .05. Analyses were conducted using SAS version 9.4 (SAS Institute, Cary, NC) statistical software.

## RESULTS

### Characteristics of Patients

**Figure 1.**
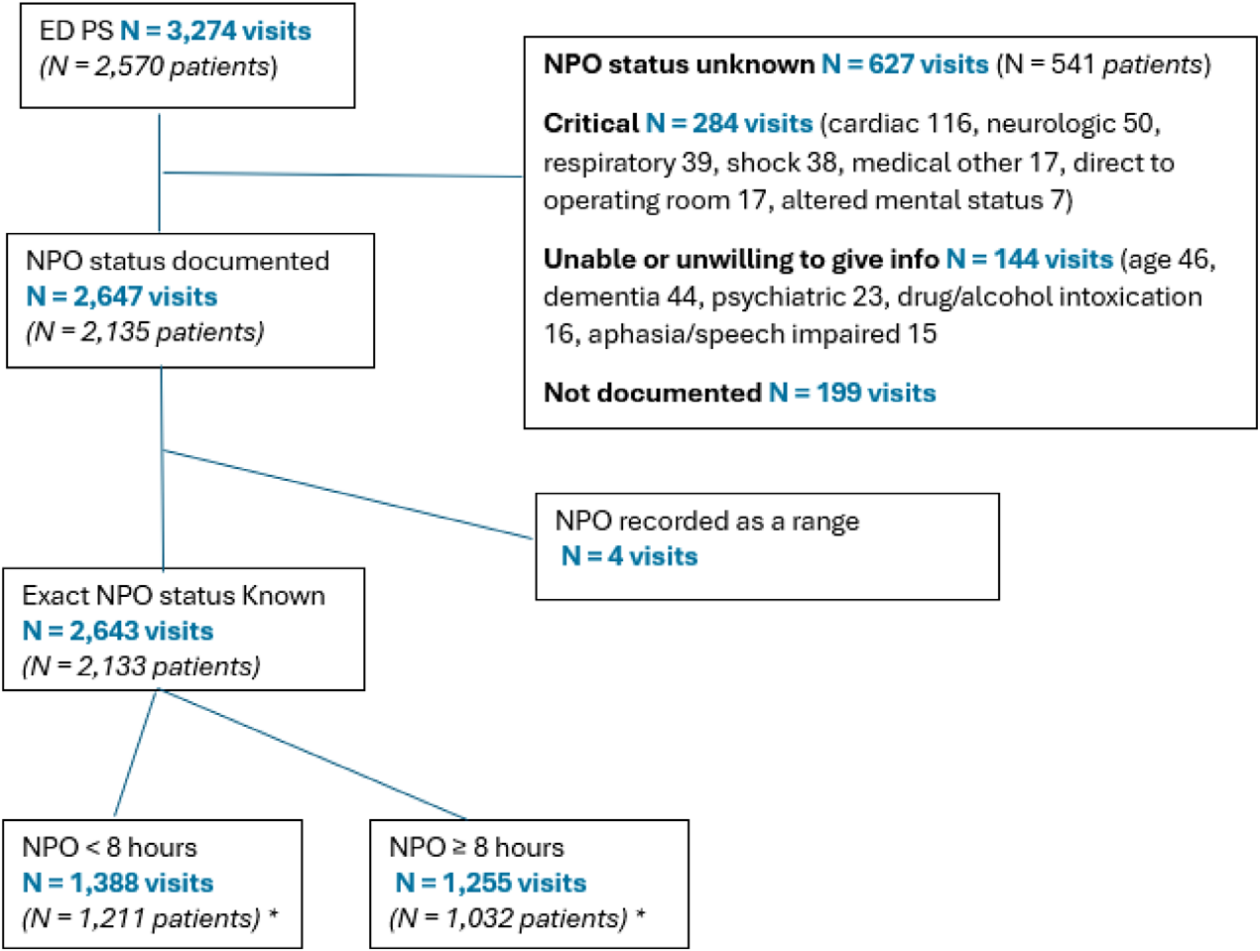
Study visit CONSORT diagram. *Number of patients does not add up to higher level total as 110 patients had multiple visits with some visits classified as NPO <8 hours and ≥8 hours and thus those patients are counted in both subgroups. Number of visits is in bold and in blue color. Number of patients is in italics and parentheses. *ED, emergecy department*; *NPO*, nil per os (nothing per mouth); *PS*, procedural sedation.

There were 3,274 PS performed in the ED on 2,570 patients of all ages by emergency physicians in the ED (Figure 1). By age group there were 1,177 PS performed on pediatric patients (age ≤21 years), and 2,097 PS performed on adults (age >22 years), of whom 708 were geriatric (≥65 years of age) PS.

### NPO Status: Known vs Unknown

The patients with NPO unknown were significantly older, with a higher acuity as denoted by higher ASA and by “more critical” procedures such as lumbar puncture, ventriculostomy, or cardioversion compared to orthopedic procedures or suturing (Table 1). When we evaluated the reasons for an unlisted NPO status these included that the patient was critical and admitted to an intensive care setting with diagnoses such as shock and/or respiratory distress or had been intubated or was undergoing emergency surgery. A significant number were unable to give reliable information about when they ate last due to medical reasons that included altered mental status, dementia, autism/developmental delay, and neurologic disorders. Another large group of patients were unwilling and/or unable to provide accurate information about their last oral intake for psychiatric reasons including acute psychiatric illness such as acute manic state or schizophrenia, or because they were experiencing substance or alcohol intoxication. There were also several young children brought in from day care or school by emergency medical services who were unaccompanied, at least initially, by an adult, such as their daycare provider or teacher or a parent, who could give information; or the adult with them had no information regarding their last oral intake. Thus, young age with lack of ability to tell time was another cause for an unknown NPO status. Additionally, some patients were only able to provide a range of time such as “more than six hours ago.” In total, there were 631 visits (19.3%) where the exact NPO was either not obtainable (13.2%) or not documented (6.1%). This emphasizes the fact that PS may need to be done in an ED without the luxury of knowing the last oral intake in about one of five patient presentations (Figure 1) (Table 1).

**Table 1.** Patient demographics, ASA and procedure characteristics.

Characteristic	Exact NPO known	Exact NPO unknown	*P*-value	NPO < 8 hours	NPO ≥ 8 hours	*P*-value
Study visits	2,643	631		1,388	1,255	
Age (years)						
Median (IQR)	37 (10–62)	53 (27–67)	< 0.001	26 (7–59)	46 (14–64)	< 0.001
Range	0.2 to 94	0.9 to 102		0.2 to 94	1.1 to 92	
Male	1500 (57%)	362 (57%)	0.78	795 (57%)	705 (56%)	0.58
Ethnicity			0.050			0.09
Black	786 (30%)	210 (33%)		431 (31%)	355 (28%)	
White	1816 (69%)	413 (66%)		931 (67%)	885 (71%)	
Other	41 (2%)	4 (1%)		26 (2%)	15 (1%)	
ASA category			< 0.001			< 0.001
ASA 1	952 (36%)	121 (19%)		589 (42%)	363 (29%)	
ASA 2	667 (25%)	160 (25%)		353 (25%)	314 (25%)	
ASA 3	904 (34%)	250 (40%)		391 (28%)	513 (41%)	
ASA 4	115 (4%)	78 (12%)		54 (4%)	61 (5%)	
ASA 5	5 (0.2%)	22 (3%)		1 (0.1%)	4 (0.3%)	
Procedure			< 0.001			< 0.001
Orthopedic procedures (total)	1610 (61%)	285 (45%)		798 (58%)	812 (64%)	
- Reduction of fracture	- 749 (28%)	- 100 (16%)		- 430 (31%)	- 319 (25%)	
- Reduction of dislocation	- 861 (33%)	- 185 (29%)		- 368 (27%)	- 493 (39%)	
Cardioversion	535 (20%)	206 (33%)		290 (21%)	245 (20%)	
Suturing/wound care	288 (11%)	38 (6%)		209 (15%)	79 (6%)	
EGD	55 (2%)	10 (2%)		19 (1%)	36 (3%)	
Lumbar puncture	35 (1%)	27 (4%)		16 (1%)	19 (2%)	
Foreign body removal	33 (1%)	4 (1%)		17 (1%)	16 (1%)	
Chest tube	16 (0.6%)	12 (2%)		5 (0.4%)	11 (0.9%)	
Hernia reduction	13 (0.5%)	7 (1%)		6 (0.4%)	7 (0.6%)	
Ventriculostomy	4 (0.2%)	13 (2%)		2 (0.1%)	2 (0.2%)	
CT scan	1 (0.04%)	8 (1%)		0 (0%)	1 (0.1%)	
Other	53 (2%)	21 (3%)		26 (2%)	27 (2%)	

ASA 1 is a normal healthy patient. ASA 2 is a patient with mild systemic disease. ASA 3 is a patient with severe systemic disease that is not life threatening. ASA 4 is a patient with severe systemic disease that is a constant threat to life. ASA 5 is a moribund patient that is not expected to survive without the operation.

*ASA*, American Society of Anesthesiology physical status; *NPO*, nil per os (nothing by mouth); *EGD*, esophagogastroduodenoscopy.

#### Adverse events and procedure completions

**Figure 2.**
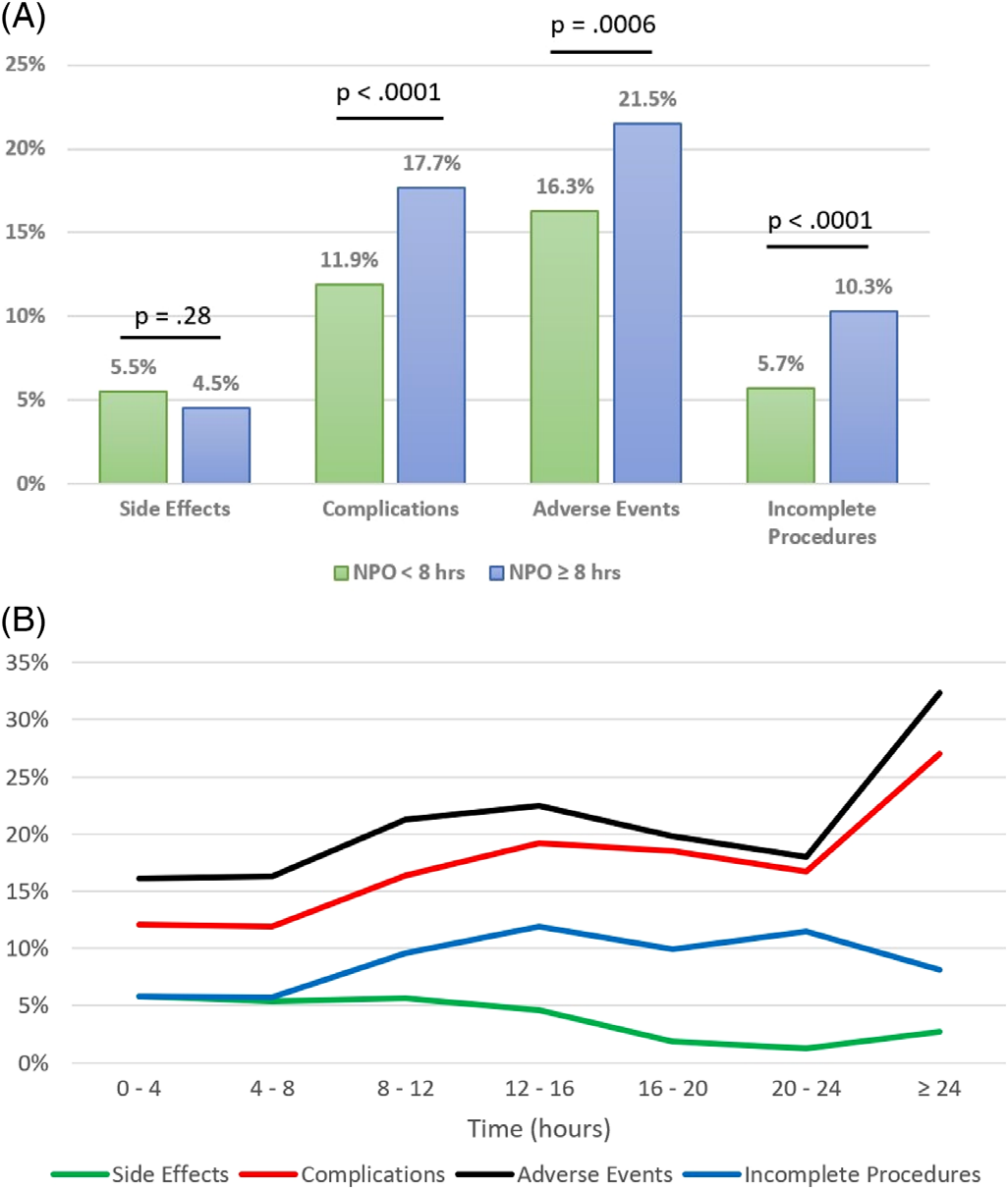
(A) Side effects, complications, adverse events, and incomplete procedures by nil per os group: <8 hours vs ≥8 hours. (B) Side effects, complications, adverse events, and incomplete procedures by nil per os. *NPO*, nil per os (nothing by mouth).

There was no significant difference for side effects, complications or adverse events between exact NPO status known vs exact NPO status unknown (Table 2). The incidence of side effects, which was primarily vomiting, was greater for NPO <8 hours at 5.5% than for NPO ≥8 hours at 4.5%, but this was not statistically significant. However, when NPO was considered numeric, it was found that as NPO time increases, the risk of a side effect, generally vomiting, significantly decreases. The complications and adverse events were significantly greater for NPO ≥8 hours than for NPO <8 hours both when NPO status was binary and numeric with complications at 11.9% for NPO <8 hours and 17.7% for NPO ≥8 hours and adverse events at 16.3% for NPO <8 hours and 21.5% for NPO ≥ 8 hours (Table 2) (Figure 2). The rate of procedural completions was significantly higher when NPO status was known (92.1% vs 86.1%) and when NPO <8 hours (94.3% vs 89.7%) (Table 2, Figure 2A). We did not find any instances of pulmonary aspiration as were noted in previous studies of ED PS.^5^ We had one intubation out of 3,274 PS (0.03%).

**Table 2.** Side effects, complications, adverse events and procedure completions according to nil per os status.

	Exact NPO status				NPO binary				NPO numeric	
	Known	Unknown	*P*-value		NPO < 8 hours	NPO ≥ 8 hours	*P*-value		Odds ratio 95% CI	*P*-value
Study visits	2,643	631			1,388	1,255			2,643	
Side effects	5.0%	4.1%	0.36		5.5%	4.5%	0.28		0.96 (0.92, 0.99)	0.02
Complications	14.6%	13.8%	0.34		11.9%	17.7%	<0.001		1.03 (1.01, 1.05)	0.001
Adverse events	18.8%	17.1%	0.25		16.3%	21.5%	<0.001		1.02 (1.004, 1.04)	0.02
Any intervention	4.2%	4.0%	0.70		4.1%	4.4%	0.75		1.01 (0.97, 1.04)	0.63
Interventions respiratory	3.4%	3.5%	0.99		3.4%	3.5%	0.90		1.01 (0.97, 1.05)	0.77
Interventions other	0.8%	0.5%	0.35		0.7%	0.9%	0.65		1.02 (0.94, 1.10)	0.64
Procedure completed	92.1%	86.1%	< 0.001		94.3%	89.7%	<0.001		0.96 (0.93, 0.98)	0.001
										
	**NPO categorized**									
	**0 to <2 hours**	**2 to < 4 hours**	**4 to < 6 hours**		**6 to < 8 hours**	**≥ 8 hours**	***P*-value**			
Study visits	31	193	474		690	1,255				
Side effects	6.5%	5.7%	5.3%		5.5%	4.5%	0.87			
Complications	16.1%	11.4%	12.9%		11.2%	17.7%	0.001			
Adverse events	19.4%	15.5%	16.9%		15.9%	21.5%	0.02			
Any intervention	0.0%	3.1%	4.9%		4.1%	4.4%	---			
Interventions respiratory	0.0%	2.6%	3.6%		3.6%	3.5%	---			
Interventions other	0.0%	0.5%	1.3%		0.4%	0.9%	---			
Procedure completed	90.3%	94.8%	93.7%		94.8%	89.7%	0.002			

*NPO*, nil per os (nothing by mouth).

Length of NPO categories is lower-bound inclusive. Thus, the first category consists of NPO values from zero to less than four hours, the second category consists of NPO values from four to less than eight, etc.

#### Interventions by NPO status

The need for intervention was relatively low overall at approximately 4%. The proportion of visits requiring intervention did not significantly differ when the exact NPO status was known (4.2%) vs unknown (4.0%) (*P* = 0.70) or when NPO <8 hours (4.1%) vs ≥8 hours (4.4%) (*P* = 0.75). Notably, there was only one intubation of 3,274 PS (0.03%). This was an elderly female with a history of hypertension undergoing reduction of a shoulder dislocation who received propofol and hydromorphone. She experienced bradypnea. She was bagged and then intubated for fewer than five minutes. She was extubated in the ED without complications and discharged home. At follow-up in the office weeks later, she was well with no problems resulting from the intubation/ED visit (Table 3).

**Table 3. tab3:** Interventions by nil per os status.

	Exact NPO known	Exact NPO unknown	NPO < 8 hours	NPO ≥ 8 hours
Study visits	2,643	631	1,388	1,255
Interventions	112 (4.2%)	25 (4.0%)	57 (4.1%)	55 (4.4%)
*Airway maneuver*				
Bag-valve mask	53 (2.0%)	16 (2.5%)	25 (1.8%)	28 (2.2%)
Non-rebreather mask	15 (0.6%)	2 (0.3%)	8 (0.6%)	7 (0.6%)
NPA	19 (0.7%)	1 (0.2%)	8 (0.6%)	11 (0.9%)
Jaw thrust/chin lift	13 (0.5%)	2 (0.3%)	6 (0.4%)	7 (0.6%)
Suctioning	4 (0.2%)	1 (0.2%)	2 (0.1%)	2 (0.2%)
Intubation	1 (< 0.1%)	0 (0%)	1 (0.1%)	0 (0%)
Airway Interventions only	91 (3.4%)	22 (3.5%)	47 (3.4%)	44 (3.5%)
*Medications*				
Medications only (including IVF)	15 (0.6%)	3 (0.4%)	9 (0.7%)	6 (0.5%)
Medications only (not including IVF)	5 (0.2%)	2 (0.3%)	5 (0.4%)	0 (0%)
IVF only	10 (0.4%)	1 (0.1%)	4 (0.3%)	6 (0.5%)
Atropine	1 (< 0.1%)	0 (0%)	1 (0.1%)	0 (0%)
Diphenhydramine	4 (0.2%)	2 (0.3%)	3 (0.2%)	1 (0.1%)
IVF	13 (0.5%)	1 (0.2%)	4 (0.3%)	9 (0.7%)
Naloxone	2 (0.1%)	0 (0%)	1 (0.1%)	1 (0.1%)
Methylprednisolone	1 (< 0.1%)	0 (0%)	1 (0.1%)	0 (0%)

*NPO*, nil per os (nothing by mouth); *NPA*, nasopharyngeal airway; *IVF*, intravenous fluids.

#### Demographic and procedural variables affecting adverse events

According to multivariate analysis, NPO status, year of visit, sex, whether transferred or not, race, and use of multiple sedatives were not significant factors associated with side effects, complications, or adverse effects. A higher ASA classification and older age group (adult/geriatric) were significantly associated with higher risk of complications and, thus, adverse events (*P* < 0.0001) but not side effects. The choice of primary sedative was significantly associated with the incidence of side effects, complications, and adverse events. Compared to propofol, the use of etomidate, ketamine, midazolam, or other sedatives all showed an increased risk of side effects, primarily vomiting; and the use of methohexital or etomidate showed a decreased risk of complications. The choice of analgesic was not associated with the incidence of side effects but was significantly associated with the occurrence of complications and adverse events. Compared to fentanyl, the use of hydromorphone, morphine, or oxycodone showed a decreased risk of complications. Using both a sedative and an analgesic was associated with a higher risk of side effects and adverse events (Table 4).

**Table 4.** Multivariate analysis of nil per os group and side effects, complications, and adverse events.

	Side effects			Complications			Adverse events		
	Odds ratio	95% CI	*P*-value	Odds ratio	95% CI	*P*-value	Odds ratio	95% CI	*P*-value
NPO < 8 hours vs. NPO ≥ 8 hours	0.93	0.64–1.34	0.68	1.09	0.86–1.39	0.48	1.13	0.92–1.39	0.26
Year	0.98	0.94–1.03	0.41	1.00	0.97–1.03	0.95	0.99	0.96–1.02	0.43
Pediatric vs adult/geriatric	1.29	0.67–2.48	0.43	0.20	0.10–0.40	<0.001	0.39	0.25–0.62	<0.001
Male vs female	1.13	0.78–1.62	0.52	0.83	0.65–1.06	0.14	0.90	0.73–1.12	0.34
Transfer	1.10	0.69–1.75	0.68	1.15	0.82–1.59	0.42	1.03	0.78–1.36	0.85
ASA	1.00	0.77–1.32	0.98	1.79	1.48–2.15	<0.001	1.55	1.33–1.81	<0.001
Race (ref = other)			0.61			0.48			0.44
Black	0.49	0.15–1.58		0.57	0.20–1.66		0.66	0.28–1.52	
White	0.47	0.15–1.52		0.65	0.23–1.85		0.73	0.32–1.66	
Primary sedative (ref = propofol)			<0.001			<0.001			0.001
Methohexital	2.40	0.93–6.16		0.52	0.29–0.93		0.60	0.36–1.00	
Etomidate	3.99	2.27–7.03		0.48	0.35–0.66		0.68	0.51–0.91	
Ketamine	4.04	2.12–7.68		1.09	0.58–2.05		1.87	1.23–2.85	
Midazolam	2.56	1.21–5.45		0.76	0.50–1.15		0.91	0.62–1.33	
Other	12.14	3.22–45.85		0.25	0.03–2.49		1.21	0.32–4.58	
Multiple sedatives	1.16	0.73–1.84	0.54	1.09	0.80–1.49	0.57	1.19	0.90–1.56	0.22
Sedative + analgesic	6.67	1.42–31.24	0.02	2.92	0.76–11.24	0.12	3.43	1.01–11.64	0.048
Primary analgesic (ref = fentanyl)			0.47			<0.001			0.007
Meperidine	0.64	0.14–2.86		1.59	0.77–3.27		1.40	0.72–2.75	
Hydromorphone	2.20	0.97–4.98		0.50	0.30–0.84		0.66	0.42–1.05	
Morphine	1.07	0.60–1.89		0.59	0.41–0.85		0.67	0.49–0.93	
Oxycodone	5.27	1.11–25.09		0.11	0.01–0.87		1.17	0.35–3.96	
Other/unknown	6.20	1.24–31.03		1.82	0.47–7.02		2.34	0.68–8.06	

Models fit are generalized estimating equations assuming compound symmetry correlation structure. ASA 1: normal healthy patient. ASA 2: mild systemic disease. ASA 3: severe systemic disease but not life-threatening. ASA 4: patient with severe systemic disease that is a constant threat to life. ASA 5: moribund patient who is not expected to survive without the operation.

*ASA*, American Society of Anesthesiology physical status; *NPO*, nil per os (nothing by mouth); *CI*, confidence interval.

#### Side effects, complications and adverse events and interventions by fasting time

Of interest was the relationship between NPO duration and patient outcomes, specifically side effects, complications, overall adverse events, any interventions, respiratory interventions, and other interventions (non-respiratory). We explored several ways of defining NPO duration (Table 2). First, we compared NPO duration status known vs unknown and found no significant difference in any patient outcome. Next, we dichotomized NPO duration as <8 hours vs ≥8 hours and found a significantly higher rate of complications and overall adverse events in the NPO ≥8 hours group. When analyzing NPO duration as a numeric variable, we saw that each additional hour of NPO was significantly associated with a decrease in risk of side effects and an increase in risk of complications and overall adverse events.

**Figure 3.**
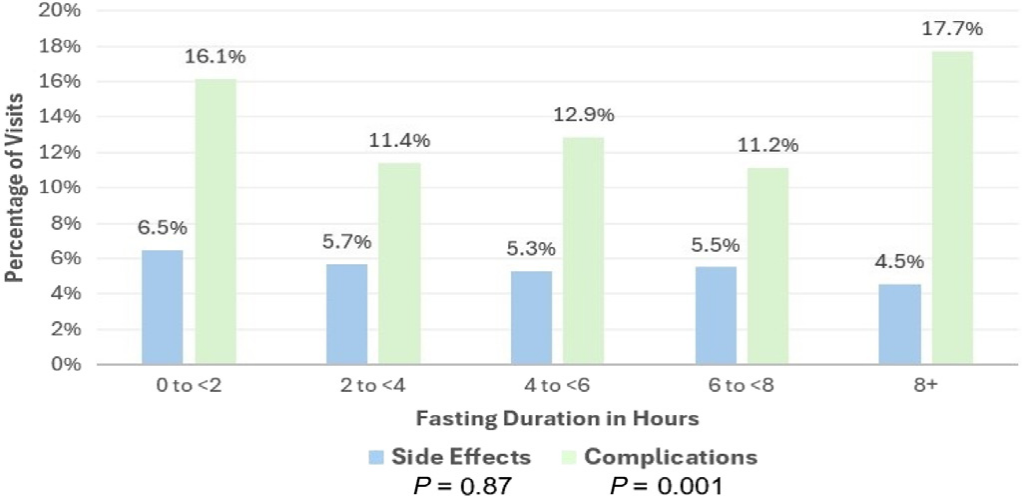
Fasting time and adverse events by fasting duration in hours.

Finally, we categorized NPO duration into two-hour intervals and compared each to the reference group of ≥8 hours. There were no significant differences in side effects when comparing the various NPO time intervals 0 to <2, 2 to <4, 4 to <6, and 6 to <8 hours to NPO ≥8 hours. Complications at NPO 2 to <4, 4 to <6, and 6 to <8 hours were significantly less than for NPO ≥8 hours. There were fewer complications in the NPO time interval 0 to <2 hours compared to NPO ≥8 hours, although this did not achieve statistical significance. However, it should be noted that the 0 to <2 hours NPO group contained only 31 visits (Figure 3, Table 2).

## DISCUSSION

Studies regarding NPO status and ED PS in the pediatric population have found no association between NPO status and adverse events.^8^ ^–^ ^13^ Surprisingly, to our knowledge, there have been no adult studies of NPO status and adverse events in ED patients from the United States and only two international studies. One small Australian study in pediatric and adult ED subjects with a somewhat atypical patient population consisting of predominately ASA 1 and 2 patients and an overwhelming majority (84%) of orthopedic patients with propofol being the only sedative found a 22.4% incidence of adverse respiratory events for not-fasted patients vs 19.5% for fasted patients and a 33.3% incidence of respiratory interventions for not-fasted compared with 24.6% for fasted patients.^16^ Our study had more than eight times the number of patient encounters than in this study and included higher acuity patients with higher ASAs, a greater range of procedures performed, and a variety of sedatives and a more robust statistical analysis that considered other potential confounding factors. Our incidence of adverse events of 16.3% for NPO <8 hours and 21.5% for those fasted ≥8 hours is comparable to the Australian study.^16^

Our overall incidence of adverse events (18.4%) compares favorably with another study, also from Australia, in pediatric and adult ED patients that looked primarily at adverse respiratory events during ED PS. Taylor et al reported a higher 20.8% incidence of just airway events and found no association of fasting status with vomiting.^17^ Our study is consistent with a prior report of elective PS in pediatric patients performed by an elective sedation service regarding predictors of complications for patients undergoing PS and another pediatric study of non-emergent sedations for research procedures, which both reported a higher rate of complications with higher ASA.^7^ ^,^ ^18^

When comparing by age group, pediatric patients had significantly fewer sedation-related complications and adverse events than adults, especially geriatric adults. Side effects, most commonly vomiting, were higher in pediatric patients than adults, which may at least be partly explained by the greater use of emetogenic sedatives, specifically ketamine, in the pediatric patients (Table 4). Ketamine as a sedative increases the risk of vomiting as compared with other sedatives.^10^ ^,^ ^11^ The specific age group—pediatric, non-geriatric adult, and geriatric adult—affected the occurrence of side effects, complications, and adverse events. This age group factor for ED PS has not been evaluated previously by robust statistical analysis (Table 4).

We found in our unadjusted analysis that fasting ≥8 hours was associated with a slightly lower incidence of side effects, mostly vomiting, and a statistically significantly greater occurrence of complications and adverse events (Table 2). This differs from the previous pediatric ED PS studies that did not find an association between adverse events and NPO status.^8^ ^–^ ^13^ In one pediatric study, there was a nonsignificant increased incidence of vomiting with increased fasting time, but the comparison was of those fasted <1 hour vs those fasted ≥3 hours.^12^ In an Australian study that included adults, there was no significant difference in the incidence of adverse procedure-related events based on the time they last ate/drank.^17^ One variable that might account for the various results could be different patient populations: pediatric vs adult patients, and varying acuity of patients, although other factors, such as medications (sedatives, analgesics) and procedure being done, could also play a role.

Our research adds to the data, primarily in the pediatric population, indicating that compliance to fasting guidelines similar to those for elective surgery does not significantly decrease the incidence of adverse events during procedural sedation. Our study documents that such adherence to the recommended fasting guidelines may result in a greater incidence of adverse events during PS. Recently, graded fasting precautions based on various factors including patient characteristics, comorbidities, the procedure, and the sedation technique have been suggested.^5^

There could be several reasons why fasting may have a negative effect. Hypoglycemia has been described in an adult diabetic patient who was fasting prior to a procedure.^19^ Patients going without PO intake may become dehydrated and hypotensive. Future studies would be helpful in collaborating whether the statistically significant negative impact of fasting prior to ED PS that we found, both on decreased PS success rates and on an increased incidence of adverse events, is specific to a certain patient population, such as higher acuity adults with multiple comorbidities and higher ASAs.

## LIMITATIONS

This study has several limitations. These results were based on the findings from one hospital system, which may limit its generalizability. However, 20 hospital EDs with many diverse locations in urban, suburban and rural settings, and varying geographic locations in two different states, were included. Moreover, we included all ages of patients with varying comorbidities, ASAs, and multiple diagnoses undergoing PS performed by many different physicians over 15.25 years using various sedatives and analgesics and had a high number (over 3,000) of ED PS.

Although this was a retrospective study, the recording of data was done prospectively at the time of the ED PS primarily by the nurses, the respiratory therapists, and the ED attending physician on a standardized four-page form used throughout the hospital on which staff has been in-serviced multiple times on a regular basis as part of the hospital-wide QI program. Data such as vital signs and rhythm strip monitoring are recorded continuously throughout all procedural sedations, and because such information is included on the form it seems highly unlikely that any adverse event such as hypo- or hypertension, bradypnea or apnea, or a low pulse oxygen saturation would not have been recorded. In addition, the ED EHR chart completed by the emergency physician in attendance during the sedation and ED visit was also reviewed. Moreover, all information was recorded prospectively, which makes it doubtful that any significant data or occurrence was missed.

Hospital policy requires that a minimum of three personnel be present throughout the ED PS: an ED registered nurse; an ED respiratory therapist; and an ED attending physician. Others such as a consultant or resident are usually present as well. This makes it improbable that this group of individuals would overlook or not record any adverse event.

Fasting was not documented in about one-fifth of patients, which could affect the validity of this study. The primary reason for this was the critical condition and higher ASA of some of our patients and the emergent nature of the procedural sedations (Figure 1). Other factors that may have contributed to missing data include the time constraints from a busy ED with high patient volumes and, perhaps, the impression that this data was not essential given the depth of sedation anticipated and the controversy over NPO status for ED PS.

Our incidence of NPO not listed is comparable with other studies. One study in a pediatric ED had fasting times not documented in 25.4% of cases, although they had younger, “healthier” patients with fewer comorbidities, lower ASAs, and fewer dangerous procedures such as ventriculostomy or cardioversion.^13^ Another study from pediatric sedation services that included scheduled sedations and sedations in non-ED settings reported 22.4% of NPO unknown.^6^

Because the NPO cutoff time of eight hours is consistently mentioned in the various guidelines and the literature, we used this eight-hour period, as well as the 2-, 4-, and 6-hour cutoff times ^2^ ^,^ ^4^ ^,^ ^7^ ^,^ ^8^ ^,^ ^13^ (Figures 2A, 3 and Tables 2 [Table tab3]–4). A recent consensus statement did not make a distinction between NPO time for solids (light meal) vs liquids (non-human milk or formula) and used the same cut-off time for all these PO intake types in healthy infants and children.^5^ Moreover, the guidelines/consensus statements have varied widely over time especially for liquid PO intake. For example, one recent guideline recommends a NPO of four hours for breast milk.^2^ Another consensus statement gives no NPO restriction for breast milk if no risk factors, two hours if some risk factors, and four hours if moderate risk factors.^5^ Another guideline also did not differentiate between solids or liquids and stated “no milk or solids after midnight.”^4^ Because of the lack of consistent NPO times,^2^ ^–^ ^5^ based on different PO intake, age, and risk factors over the years, particularly for PO liquid intake, and the lack of differentiation between solids and liquids in various guidelines/consensus statements,^4^ ^,^ ^5^ we used NPO for any PO intake in our analysis.

Observers were not blinded to the medications administered or fasting times, which could have led to bias. However, observers were unaware of this study. Our sedation form has a blank for the time of last PO intake but does not specify whether liquids or solids were consumed, although this was recorded in some instances.

## CONCLUSION

To our knowledge this is the largest ED procedural sedation cohort that included adults, particularly geriatric patients and higher acuity patients, analyzed with the most robust statistical analyses to evaluate the association among nil per os status and adverse events. We identified a significant increase in complications and adverse events and incomplete procedures for those NPO ≥8 hours vs NPO <8 hours. These results indicate that delaying sedation to meet established fasting guidelines may worsen outcomes for patients of all ages, including adults in the ED, and is not indicated.
